# Metabolism and cancer: the future is now

**DOI:** 10.1038/s41416-019-0667-3

**Published:** 2019-12-10

**Authors:** Christian Frezza

**Affiliations:** MRC Cancer Unit, University of Cambridge, Cambridge, UK

## Abstract

In the last decade, the field of cancer metabolism transformed itself from being a description of the metabolic features of cancer cells to become a key component of cellular transformation. Now, the potential role of this field in cancer biology is ready to be unravelled.

## Background

If we look back 10 years ago, the main questions in the field of cancer metabolism were whether and why cancer cells exhibited the so-called “Warburg effect”, a metabolic switch from oxidative to glycolytic metabolism. For years it was unclear why fast proliferating cancer cells would undergo this metabolic switch considered by many an inefficient use of nutrients (reviewed in ^1^). This fervid discussion was epitomised by a landmark review from Vander Heiden and colleagues,^2^ who proposed that the “Warburg effect” should not be viewed solely from the point of view of energy generation, but, instead, should be considered as a mean to synthesise anabolic molecules. This change in perspective, almost a new Weltanschauung for the field, fostered new lines of enquiries to determine how cancer cells fulfil their biosynthetic needs, and connected the “Warburg effect” to nucleotide, lipids, and protein metabolism. Nowadays, we are facing another revolution. After years spent charting the many metabolic pathways explored by cancer cells to retrieve nutrients for growth and proliferation, the community has realised that the metabolic layer is at the interface between many other cellular processes in the cells and that it is more heterogeneous and subject to external cues than anticipated. In this second instalment of the special issue on Cancer Metabolism in the *British Journal of Cancer*, which has been co-edited by me and Professor Adrian Harris, we present primary research papers and reviews that investigate poorly explored areas of cancer metabolism and the many processes to which dysregulated metabolism contributes.

## Mapping cancer metabolism

Despite the many recent efforts, the metabolic reprogramming of cancer cells is far from being fully characterised because of technical and experimental limitations. In this special issue, several contributions added essential pieces to this puzzle. For instance, Berndt et al.^3^ capitalise on a unique in silico modelling approach using proteomics data not only to predict metabolic changes in liver cancer, but also to identify metabolic pathways whose inhibition selectively affects cancer cells. In addition, Becker^4^ provides an extensive review of the regulation of pH in cancer cells and proposes the concept of a ‘transport metabolon’, whereby multiple transporters act together to regulate acid/base homoeostasis in cancer cells, a key regulator of cellular metabolism.

The dysregulation of mitochondrial function remains one the main components of the metabolic reprogramming of cancer. Here, Raimondi and colleagues^5^ provide a comprehensive review of the connection between dysregulation of electron transport chain and cancer, focusing on the formation of reactive oxygen species (ROS), whose role in cancer biology has never been more debated. They propose that if, on one hand, multiple oncogenic cascades can cause aberrant ROS production, ROS production itself can trigger oncogenic processes, making it very difficult to disentangle the cancer-causing role of oxidative stress. Ciccarone and colleagues^6^ demonstrate that the mitochondrial enzyme aconitase 2 (ACO2) is reduced in breast cancer and, when overexpressed, it can dysregulate pyruvate metabolism, revealing a potential metabolic vulnerability in cancers associated with ACO2 loss. Zhang et al.^7^ show that the modulation of mitofusin 1, a protein involved in mitochondrial fusion, can also affect cellular metabolism with implications for cancer biology. Indeed, they found that in hepatocellular carcinoma, MFN1 is suppressed and its loss leads to defects in mitochondrial metabolism and promotes metastasis.

## Cancer metabolism is heterogeneous and subject to environmental cues

The metabolic reprogramming of cancer is by and large transcriptionally regulated by oncogenes and mutated tumour suppressors. Yet, it is now clear that the metabolic composition of the tumour microenvironment can affect the metabolic phenotype of the cells. Therefore, cancer cells in a tumour, exposed to different levels of oxygen and nutrients, might be metabolically heterogeneous and their metabolic phenotype could further change during tumour progression, when nutrients become limiting. In this special issue, Nanda et al.^8^ provide a comprehensive description of the genetic and environmental cues that drive cancer metabolism, with a specific focus on the cell-intrinsic and cell-extrinsic factors that contribute to metabolic heterogeneity. Importantly, as highlighted by Vettore and colleagues,^9^ cancer cells and other components of the tumour microenvironment, including fibroblasts and immune cells, form a metabolic community and exchange metabolites regulating each other’s functions. Intriguingly, metabolites within the tumour microenvironment can affect cancer cell behaviour beyond providing energy substrates. For instance, Sola-Penna and colleagues^10^ have identified the neurotransmitter serotonin as an important player in cell-to-cell communication mediated by the serotonin receptor. They then show that serotonin signalling offers a proliferative advantage to breast cancer cells by both increasing cell proliferation and decreasing cell death.

Technical limitations of metabolomic analyses, which often require the destruction of the tumour tissue, do not allow a full characterisation of tumour heterogeneity. To overcome these issues, new technologies are becoming available to analyse metabolite levels in intact tissues. Björkblom and colleagues^11^ describe the use of microdialysis to investigate the metabolic changes in brain tumours upon treatment with the anticancer drug cisplatin, highlighting distinct metabolic patterns associated with the treatment. In another paper, Kawashima and colleagues^12^ describe the use of matrix-assisted laser desorption and ionisation imaging mass spectrometry to investigate the distribution of phosphatidylinositols in human tumours. They found a correlation between the accumulation of these metabolites and invasion and nodal metastasis in breast cancer.

## Targeting metabolism

A vital outcome of the work in cancer metabolism is our ability to translate these findings into actionable anticancer targets. In this special issue, several primary research papers provide important examples to support that targeting metabolism can be used in the clinic. For instance, James et al.^13^ show that the inhibition of the cancer-specific pyruvate kinase 2 in pancreatic cancer cells using shikonin reduces their growth and invasion. Intriguingly, though, they demonstrate that this toxicity is due to the inhibition of a pool of PKM2 localised at the plasma membrane, where it provides a privileged ATP supply to the ATP-dependent plasma membrane calcium pump. Therefore, the inhibition of PKM2 causes calcium overload and cell death, rather than the expected metabolic catastrophe. In a different context, breast cancer, Lord and colleagues^14^ elegantly showed that metformin, a drug commonly used in Type 2 diabetes but with promising yet unclear anticancer effects, acts as an inhibitor of fatty acid oxidation at clinical doses. This work, together with their previous finding that the mitochondrial response to metformin in primary breast cancer defines antitumour effect,^15^ suggests that metabolic profiling can be used to predict the response to metformin, and more in general to anticancer drugs. Finally, Liu and colleagues^16^ describe the role of cystine metabolism in cancer cells and report that the activation of the trans-sulphuration pathway to which cysteine contributes plays a key role in the resistance to a specific iron-dependent form of cell death defined ferroptosis.

## Conclusions

In summary, many discoveries were made in this last decade in the field of cancer metabolism. Never before have we achieved such a comprehensive understanding of the metabolic needs of cancer cells. We have learned that this metabolic rewiring is not only needed to fuel energy needs or to support biomass generation, but it has essential roles in other cancer features such as migration, invasion, and metastasis. We have also evidence that targeting some of these metabolic pathways can reduce cancer growth if not as a single agent, probably in combination with other anticancer drugs. Yet, we should not be complacent. Indeed, emerging evidence indicates that cancer metabolism is highly dynamic and heterogenous, and the current analytical platforms do not enable us to grasp this complexity in full. Better technologies to investigate metabolism at the single-cell level without disrupting the tumour tissue are needed. If the progress is as steady as the one we experience in the last decade, I have no doubts we can break this barrier and achieve an even deeper understanding of the role of metabolism in cancer.

## Data Availability

Not applicable.
